# Incontinence outcomes in women undergoing retropubic mid-urethral sling: a retrospective cohort study comparing Safyre™ and handmade sling

**DOI:** 10.1590/S1677-5538.IBJU.2021.0646

**Published:** 2022-03-18

**Authors:** Fernando Terziotti, Emerson Pereira Gregório, Marcio Augusto Averbeck, Silvio Henrique Maia Almeida

**Affiliations:** 1 Programa de Pós-Graduação em ciências da saúde Universidade Estadual de Londrina Londrina PR Brasil Programa de Pós-Graduação em ciências da saúde da Universidade Estadual de Londrina - UEL, Londrina, PR, Brasil;; 2 Escola de medicina Pontifícia Universidade Católica do Paraná Londrina PR Brasil Escola de medicina, Pontifícia Universidade Católica do Paraná - PUCPR, Londrina, PR, Brasil;; 3 Departamento de Urologia Hospital Moinhos de Vento Porto Alegre RS Brasil Departamento de Urologia, Hospital Moinhos de Vento, Porto Alegre, RS, Brasil;; 4 Departamento de Cirurgia Universidade Estadual de Londrina Londrina PR Brasil Departamento de Cirurgia, Universidade Estadual de Londrina - UEL, Londrina, PR, Brasil

**Keywords:** Urinary Incontinence, Suburethral Slings, Postoperative Complications

## Abstract

**Purpose:**

This study examined and compared efficacy, safety, satisfaction, and complications of the retropubic Safyre™ sling and a retropubic hand-made synthetic sling (HMS) in a short-, mid- and long-term follow-up.

**Methods:**

We retrospectively reviewed a prospectively maintained database of women who underwent Safyre™ or HMS between March 7^ths^ 2005 and December 27^ths^, 2017. Patients had first assessment (7-10 days), second (40-45 days), and third (sixth month) postoperatively. Between September and December 2018, patients who completed at least one year of surgery, received a telephone call. Follow-up compared quartiles of follow-up time to determine complications (Clavien-Dindo), success rates (International Consultation on Incontinence Modular Questionnaire for Urinary Incontinence Short Form – ICIQ-UI SF), and patient satisfaction.

**Results:**

Three hundred fifty-one patients underwent surgery and 221 (63%) were evaluated after a median of 78.47 (± 38.69) months, 125 (55%) in the HMS, and 96 (45%) in the Safyre™ group. Higher intraoperative bladder injury was observed with Safyre™ (0% vs. 4.2%, p=0.034), and a tendency for urinary retention, requiring indwelling urinary catheter over 24 hours (2.4% vs. 8.3%, p=0.061). Both HMS (p<0.001) and Safyre™ (p<0.001) presented improvements on ICIQ-UI SF. There were no differences in satisfaction, subjective cure rates, ICIQ-UI SF, or complications between groups.

**Conclusions:**

Both HMS and Safyre™ have similar satisfaction and subjective cure rates, with marked ICIQ-UI SF score improvement. Higher rates of intraoperative bladder injury were seen in patients who received Safyre™ retropubic sling.

## INTRODUCTION

Midurethral synthetic slings (MUS) became the most commonly performed minimally-invasive procedure for treating female stress urinary incontinence (SUI) worldwide ( 1 , 2 ) and are regarded as the gold standard surgical treatment ( 3 , 4 ). There are different types and brands of commercial synthetic slings, each with their own characteristics, which can be related to distinct complications ( 5 - 7 ).

However, commercial kits can be expensive, especially in developing countries, restricting their access, particularly in the public health system ( 8 - 12 ). Therefore, low-cost hand-made synthetic slings (HMS) have been proposed as an alternative to expand their access ( 8 - 12 ).

Safyre™ is a hybrid tape developed as a re-adjustable sling ( 11 ). It is based on the fibrotic encapsulation induced by the silicone columns and allows the anchoring tails moving up or downwards ( 13 , 14 ).

Our hypothesis is that the HMS has a similar performance in comparison to the Safyre™ sling. This study aimed to present efficacy, safety, satisfaction, and complications of the retropubic Safyre™ sling vs HMS for female SUI in a long-term follow-up.

## MATERIALS AND METHODS

This study protocol was submitted and approved by local ethics committee (number 223/2009 from May 5^th^, 2010). We retrospectively reviewed a prospectively maintained database with SUI patients over 18 years of age who underwent retropubic Safyre™ or retropubic HMS between March 7^ths^ 2005, and December 27^ths^ 2017, performed by a single surgeon, with a minimum follow-up of 1 year postoperatively. Patients with urgency-predominant mixed urinary incontinence, neurogenic lower urinary tract dysfunction, and pelvic organ prolapse (grade ≥ 2) were excluded. Baseline assessment included a detailed clinical history, urogynecological examination, urodynamic evaluation, and International Consultation on Incontinence Modular Questionnaire for Urinary Incontinence Short Form (ICIQ-UI SF) ( 15 ).

Women were allocated in two groups: retropubic hand-made sling (HMS) or retropubic Safyre™ (commercial kit – *Promedon, Cordoba, Argentina* ). The choice for each of the groups was based on the mesh availability at the institution during the study period. A routine medical counseling provided patients with information about different treatment options, pros, and potential cons of these treatments. All patients received a comprehensive guidance concerning distinct surgical techniques, including synthetic and autologous slings, and Burch colposuspension procedure. Risks and benefits of different approaches were included in informed consent form. The medical team explored ideas, expectations, fears, and motivations of patients, aligning their expectations regarding the surgical treatment.

Safyre™ consists of a 100g/m2 monofilament and macroporous polypropylene mesh as suburethral support, measuring 42mm long and 13mm wide, connected to two solid polydimethylsiloxane (silicone) elastomer fixation arms, which allow adjustment of the tension of the mid-urethral sling intra and postoperatively ( 14 , 16 ).

The HMS was performed with a standardized technique by cutting a 80mm long and 15mm wide rectangle from monofilament and macroporous polypropylene mesh (Parietene™ Standard – *Medtronic, Minneapolis, USA* ) 75g/m2, attached with polyglycolic acid sutures at its edges ( 10 ), and using a resterilized Safyre™ needle. The 15mm-wide cut had the objective of leaving the mesh edge with complete braiding, and without denting, to maintain its integrity and reduce tissue damage during its traction. Sutures were passed through into the needles for retropubic positioning and no additional sutures were used for fixation.

Surgical steps and materials, including needles, were the same for both groups. Spinal anesthetic block was performed and 2g of prophylactic cefazolin was administered. A vertical 3 cm incision was made in the anterior vaginal wall and the periurethral space was then dissected. Needles were passed retropubic (upside-down) through a 1cm suprapubic incision and the sling allocated under the middle urethra without tension ( 17 ). Urethrocystoscopy was performed at the end of the procedure. Indwelling urinary catheter was maintained for 12 to 24 hours. After spontaneous voiding, patients were discharged. Any post-operative readjustment was performed in the operating room (loosening and tightening) before hospital discharge, similarly, to proposed by Toledo et al. ( 18 ).

First in-person, postoperative medical assessment was performed 7-10 days after surgery, second after 40-45 days, and the third in the sixth postoperative month. Patients with any complications were revaluated in-person even after this period. Postoperative assessments aimed to recognize complications, such as urinary retention, voiding and storage lower urinary tract symptoms (LUTS), persistent incontinence, *de novo* urgency, hematoma, persistent pain, bleeding, vaginal discharge (any bleeding intensity referred by women), urinary tract infection, vaginitis, dyspareunia or hispareunia, mesh extrusion, and macroscopic hematuria. Postoperative urinary retention was evaluated in-person at office until the sixth postoperative month and considered when ultrasound demonstrated > 100mL of post void residual volume. Complications were reported according to Clavien-Dindo classification.

Medical records were reviewed between September and December 2018, and patients who had completed at least one year of surgery, received a telephone call from a trained researcher (who was blinded to the sling subtype). During the telephone evaluation, subjective cure (defined as the absence of SUI reported by the patient) and overall satisfaction with surgery (classified dichotomously as satisfied or unsatisfied) were assessed. ICIQ-UI SF was also reapplied, and patients were asked about voiding symptoms, SUI, urgency and urgency urinary incontinence, chronic pain, and dyspareunia. Medical records were additionally reviewed in search for additional surgical procedures, medical treatments, and other unrelated complications.

P-value <0.05 was considered statistically significant, with a 95% confidence interval (CI). Initially, the Kolmogorov-Smirnov test was applied to evaluate the normal distribution of continuous variables. Continuous variables with normal distribution are reported as means and standard deviations and those without normal distribution as medians and interquartile variation. Categorical variables are presented in frequencies and percentages. Differences between HMS and Safyre™ groups were evaluated by the T-test for independent samples for continuous variables with normal distribution; the Mann-Whitney U test for continuous variables without normal distribution; and for categorical variables, the chi-square test or Fisher’s exact test, when samples were small (20<n<40 and expected frequency <5). Paired samples were analyzed by the Wilcoxon test. Chi-square test for trend was used to compare ordinal variables. Satisfaction rate, subjective cure, and ICIQ-UI SF were compared between the groups after patients had been divided into quartiles according to the period of follow-up.

## RESULTS

**Figure 1:**
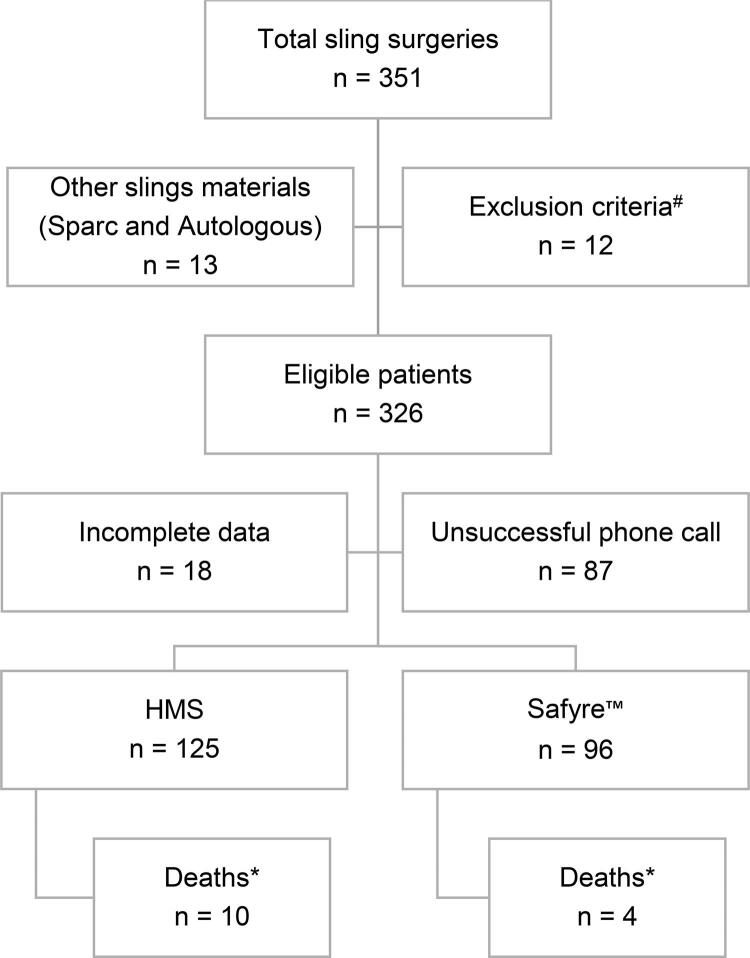
Flowchart of study selection process HMS: Handmade Sling *Deaths occurred at follow-up due to other causes (neoplasm, trauma, diverticular disease); data were used only for preoperative evaluations ^#^Neurogenic bladder, previous bladder augmentation, pelvic organs prolapse (grade ≥ 2)

A total of 351 patients underwent surgical treatment between 2005 and 2017, and 221 (63%) were able to complete the study protocol, with follow-up ranged from 13 to 165 months ( Figure-1 ). Mean age was 59.55 (±11.89) years, ranging from 31 to 84 years. Mean follow-up was 78.47 (±38.69) months, ranging from 13 to 165 months. Number of deaths in this period was 14 (10 in the HMS group and 4 in the Safyre™ group [p=0.246]), not related with the procedure (2 of metastatic breast cancer, 1 of lung cancer, 1 of leukemia, 1 due car accident, 8 of cardiovascular disease, 1 due diverticular disease).

There were no statistical differences between groups in relation to age, body mass index (BMI), previous surgical procedures (synthetic and autologous slings or Burch colposuspension procedure), hormonal status, number of pregnancies, birth routes, comorbidities (hypertension, obesity, depression, diabetes mellitus, chronic obstructive lung diseases, smoking, and congestive heart failure), and urodynamic data. Safyre™ patients had higher percent use of daily pads before surgery (60.4% vs. 40%, p=0.004), but the preoperative ICIQ-UI SF was similar in both groups (p=0.164) ( Table-1 ).

**Table 1:** Baseline data and urodynamic profile of patients according to the type of sling performed.

		HMS n=125 (56%)	Safyre™ n=96 (44%)	p-value
**Age (years), mean (SD)**		58.94 (±11.62)	60.33 (±12.26)	0.391^b^
**Concomitant surgery**		33 (26.4%)	26 (27.1%)	0.909^a^
**BMI (kg/m** ^**2**^ **), mean (SD)**		28.38 (±5.19)	27.69 (±4.64)	0.307^b^
Hormonal Status	Menopause without HR	81 (64.8%)	48 (50.0%)	0.149^d^
	Menopause with HR	18 (14.4%)	27 (28.1%)	
	Premenopausal	26 (20.8%)	21 (21.9%)	
Pads usage	Daily	50 (40.0%)	58 (60.4%)	**0.004** ^**a**^
	If necessary	25 (20.0%)	18 (18.8%)	
	None	50 (40.0%)	20 (20.8%)	
**Preoperative ICIQ-UI SF, median (IR)**		10 (9.0 – 11.0)	10 (9.0 – 12.0)	0.164^c^
Parity, median (IR)	Pregnancies	3.0 (2.0 – 3.25)	2.0 (2.0 – 3.0)	0.156^c^
	Normal birth	2.0 (0 – 3.0)	2.0 (0 – 3.0)	0.264^c^
	Cesarean section	0 (0 – 1.25)	1.0 (0 – 2.0)	0.330^c^
Previous surgeries	Incontinence	19 (15.2%)	17 (17.7%)	0.617^a^
	Pelvic Organ Prolapse	39 (31.2%)	23 (24.0%)	0.235^a^
	Hysterectomy	34 (27.2%)	26 (27.1%)	0.985^a^
	Abdominal (others)	23(18.4%)	19 (19.8%)	0.794^a^
**VLPP (cmH**_**2**_ **0), mean (SD)**		103.48 (±38.01)	103.04 (±40.83)	0.934^b^
Leakage type	Stress	111 (88.8%)	82 (85.4%)	0.453^a^
	Mixed	14 (11.2%)	14 (14.6%)	
Post-voiding residual urine	<30mL	93 (74.4%)	82 (85.4%)	0.665^d^
	30-100mL	21 (16.8%)	4 (4.2%)	
	>100mL	11 (8.8%)	10 (10.4%)	

**HMS** = Handmade Sling; **BMI** = Body Mass Index; **HR** = Hormonal Replacement; **ICIQ-UI SF** = International Consultation on Incontinence Modular Questionnaire for Urinary Incontinence Short Form; **VLPP** = Valsalva leak-point pressure; **SD** = standard deviation; **IR** = interquartile range

^a^ Chi-squared test; ^b^ T-Test; ^c^ Mann-Whtney U test; ^d^ Chi-squared test for trend; ^e^ Fisher’s exact test

Intraoperative bladder injury was higher in the Safyre™ group (0% vs. 4.2%, p=0.034). There was a tendency for urinary retention, requiring indwelling urinary catheter over 24 hours in the Safyre™ group (2.4% vs. 8.3%, p=0.061) ( Table- 2). In the HMS group, 7 patients (5.6%) required mesh adjustment (loosening: 3, tightening: 1, and urethrolysis: 3), while in the Safyre™ group, a total of 8 patients (8.3%) needed readjustments (loosening: 2, tightening: 2, and urethrolysis: 4) (p=0.434). Medical records at the sixth postoperative month revealed no differences between groups regarding SUI, urgency incontinence, mixed leakage, or pain ( Table-2 ). All observed vaginal extrusions were on the suture line, smaller than 1 cm, and without infection. Extrusions occurred in 12.8% vs. 6.2%, (p=0.107), respectively in the HMS and the Safyre™ groups, initially treated with topical estrogen therapy and, if unsuccessful, with partial extruded mesh removal under local anesthesia. One patient in the Safyre™ group presented bladder erosion requiring surgical removal ( Table-2 ). Median onset of vaginal extrusion was 6.0 (2.0 - 12.0) months in the HMS group and 3.0 months (2.0 – 4.0) in the Safyre™ group (p=0.120). There were no differences between groups for Grade II (3.2% vs. 2.1%), IIIa (4.0% vs. 3.1%), and IIIb (12.0% vs. 8.3%) Clavien-Dindo complications, respectively (p=0.282) ( Table-2 ).

**Table 2:** Complications according to the type of sling performed.

		HMS n=125 (56%)	Safyre™ n=96 (44%)	p-value
				
Intraoperative bladder injury		0 (0.0%)	4 (4.2%)	**0.034** ^**e**^
Urinary infection		14 (11.2%)	10 (10.4%)	0.853^a^
Vaginal bleeding		28 (22.4%)	18 (18.8%)	0.508^a^
LIC>24h		3 (2.4%)	8 (8.3%)	0.061^e^
Sling readjustment	None	118 (94.4%)	88 (91.7%)	0.434^e^
	Tightening ^g^	1 (0.8%)	2 (2.4%)	
	Loosening ^g^	3 (2.4%)	2 (2.1%)	
	Urethrolysis ^h^	3 (2.4%)	4 (4.2%)	
**6th month office reassessment**				
Stress Incontinence		12 (9.6%)	11 (11.5%)	0.654^a^
Urgency Incontinence		23 (18.4%)	24 (25.0%)	0.235^a^
Mixed Incontinence		5 (4.0%)	10 (10.4%)	0.060^a^
Pain		24 (19.2%)	14 (14.6%)	0.367^a^
**Vaginal extrusion**		16 (12.8%)	6 (6.2%)	0.107^a^
Time (months), median (IR)		6.0 (2.0 – 12.0)	3.0 (2.0 – 4.0)	0.120^c^
Treatment	None	109 (87.2%)	90 (93.7%)	0.118^e^
	Topical	12 (9.6%)	4 (4.2%)	
	Surgical	4 (3.2%)	2 (2.1%)	
Clavien-Dindo	None	101 (80.8%)	83 (86.5%)	0.282^e^
	II	4 (3.2%)	2 (2.1%)	
	IIIa	5 (4.0%)	3 (3.1%)	
	IIIb	15 (12.0%)	8(8.3%)	

**HMS** = Handmade Sling; **LIC>24h** = Long-term indwelling catheter for more than 24 hours.

**IR** = Interquartile Range

^a^ Chi-squared test; ^c^ Mann-Whitney U test; ^e^ Fisher’s exact test; ^g^ Performed before discharged; ^h^ Performed before sixth postoperative month

Mean follow-up was longer in the HMS group (85.05 vs. 69.90 months, p=0.004), but there were no differences between groups regarding complications, patient satisfaction, and median pre and postoperative ICIQ-UI SF. ICIQ-UI SF scores showed improvements from pre to postoperative measurements in both HMS (10 vs. 3, p<0.001) and Safyre™ (10 vs. 3.5, p<0.001) groups. Loss of follow-up was similar in both groups (p=0.163) ( Table-3 ). Tables 4 an 5 (supplementary files) present additional information about the ICIQ-IU SF scores (pre and postoperatively), according to follow-up quartiles, between sling groups.

**Table 3:** Telephone evaluation according type of sling performed.

	HMS n=115 (56%)	Safyre™ n=92 (44%)	p-value
Deaths*	10 (8.0%)	4 (4.2%)	0.246^a^
Loss of follow-up	58 (31.7%)	31 (24.4%)	0.163^a^
Follow-up (months), mean (SD)	85.05 (±40.93)	69.90 (±33.90)	0.004^b^
Postoperative ICIQ-UI SF, median (IR)	3 (0.0 –12.75)	3.5 (0.0 – 9.5)	0.476^c^
ICIQ-UI SF dif (pre – post), median (IR)	6 (2 – 8)	6 (4 – 8)	0.142^c^
De novo urgency	46 (40.0%)	39 (42.4%)	0.728^a^
Urinary Tract Infection	20 (17.4%)	17 (18.5%)	0.839^a^
Vaginal bleeding	3 (2.6%)	0 (0.0%)	0.256^e^
Voiding symptoms	13 (11.3%)	4 (4.3%)	0.070^a^
Pelvic pain	8 (7.0%)	5 (5.4%)	0.654^a^
Dyspareunia	0 (0.0%)	3 (3.3%)	0.086^e^
Satisfaction	91 (79.1%)	76 (82.6%)	0.529^a^
Subjective Cure	79 (68.7%)	73 (79.3%)	0.085^a^

**HMS** = Handmade Sling; **ICIQ-UI SF** = International Consultation on Incontinence Modular Questionnaire for Urinary Incontinence Short Form; **dif** = difference.

**SD** = Standard Deviation; **IR** = Interquartile Range;

a Chi-squared test; b T-test; c Mann-Whitney U test; e Fisher’s exact test

* Not related to the procedure

**Table 4:** Median (IR) of preoperative and postoperative ICIQ-UI SF according to the follow-up quartiles and type of sling performed.

	HMS		

Follow-up (months)	Preoperative ICIQ-UI SF	Postoperative ICIQ-UI SF	p-value
13 – 42 (n=27)	9 (8.5 – 12)	0 (0 – 12)	**0.002** ^**f**^
43 – 79 (n=21)	10 (9 – 12)	9 (0 – 16)	0.163f
80 – 103 (n=28)	10 (9 – 10.5)	3 (0 – 13.5)	**0.005** ^**f**^
104 – 165 (n=39)	9 (8 – 12)	5 (0 – 13)	**0.008** ^**f**^

**Total (n=115)**	10 (9 – 11)	3 (0 – 13)	**<0.001** ^**f**^

	Safyre™		

13 – 42 (n=24)	9.5 (8.25 – 11)	1.5 (0 – 8)	**0.006** ^**f**^
43 – 79 (n=31)	10 (8.5 – 13)	5 (0 – 13)	**0.002** ^**f**^
80 – 103 (n=24)	11 (8.5 – 13.5)	5 (0 – 8)	**<0.001** ^**f**^
104 – 165 (n=13)	9.5 (9 – 12.5)	3 (0 – 11)	**0.007** ^**f**^

**Total (n=92)**	10 (9 – 12)	3.5 (0 – 9.75)	**<0.001** ^**f**^

**HMS** = Handmade Sling; **ICIQ-UI SF** = International Consultation on Incontinence Modular Questionnaire for Urinary Incontinence Short Form.

**IR** = Interquartile Range

^f^ Wilcoxon test

**Table 5:** Comparison of difference in ICIQ-UI SF (pre – post), satisfaction, and subjective cure, according to follow-up quartiles, between sling groups.

Follow-up (months)	HMS	Safyre™	p-value
**ICIQ-UI SF difference (pre – post), median (IR)**			
13 – 42 (n=51)	8 (-2.25 – 9) (n=27)	7 (1.5 – 10) (n=24)	0.813^c^
43 – 79 (n=52)	3 (-4.25 – 10) (n=21)	5 (-0.5 – 9) (n=31)	0.386^c^
80 – 103 (n=52)	7 (-2.5 – 9) (n=28)	6.5 (2 – 10.5) (n=24)	0.138^c^
104 – 165 (n=52)	6 (-3 – 9) (n=39)	6 (2.5 – 9) (n=13)	0.336^c^
**Satisfaction, n (%)**			
13 – 42 (n=51)	24 (89%)	22 (91.7%)	0.890^a^
43 – 79 (n=52)	12 (57.2%)	27 (87.1%)	**0.034** ^**a**^
80 – 103 (n=52)	21 (75%)	18 (75%)	0.748^a^
104 – 165 (n=52)	34 (87.2%)	9 (69.2%)	0.290^a^
**Subjective Cure, n (%)**			
13 – 42 (n=51)	21 (77.8%)	20 (83.3%)	0.884^a^
43 – 79 (n=52)	14 (66.7%)	25 (80.6%)	0.414^a^
80 – 103 (n=52)	19 (67.9%)	20 (83.3%)	0.335^a^
104 – 165 (n=52)	25 (64.1%)	8 (61.6%)	0.868^a^

**HMS** = Handmade Sling; **ICIQ-UI SF** , International Consultation on Incontinence Modular Questionnaire for Urinary Incontinence Short Form. **IR** = Interquartile Range

^a^ Chi-squared test; ^c^ Mann-Whitney U test

## DISCUSSION

To our knowledge, this is the first study describing HMS vs retropubic SafyreTM sling outcomes at long-term follow-up. Analysis demonstrated similar satisfaction, subjective cure rates, and improvement in the ICIQ-UI SF in up to 13 years follow-up comparing SafyreTM with a retropubic handmade sling. Patients undergoing SUI surgery using HMS or Safyre™ retropubic slings presented similar satisfaction and subjective cure rates. Perioperative bladder injuries were more frequent in the Safyre™ group, besides a higher tendency for indwelling bladder catheterization. Loss of follow-up in the study (37%) may have been seen as an inherent limitation, but similar rates have been previously reported by Kuprasertkul and Zimmern, who demonstrated rates of loss ranging from 10 to 49% over 10 years follow-up ( 19 ).

A limited number of publications have studied Safyre™ and all of them used a transobturator approach, with bladder perforation rates varying between 0 – 4.2% ( 1 , 9 , 10 ). Risk factors for bladder injury in this context may include previous pelvic surgeries (cesarean section, colposuspension, rectocele), inexperienced surgeons, local anesthesia, younger patient age and lower body mass index (BMI) ( 4 , 20 ). In the current study, previous surgeries and BMI were similar in both groups. Additionally, the surgeon had extensive experience with both slings, and the needles were the same for both groups. Although our study design does not allow us to understand the exact mechanism behind higher rates of intraoperative bladder perforation in the Safyre™ group, since cystoscopy was performed at the end of the procedure, further research should focus on the design of this sling, particularly on its solid elastomer (silicone) fixation arms. Kuschel S and Schuessler B, in a prospective trail, founded vaginal sling extrusion in 8.8% of the patients and a pre-erosive state in another 13.9% (concerning the central polypropylene part). The lateral silicone column could be palpated medial to the pubic bone in 47% of the patients indicating dislocation ( 13 ).

The retropubic Safyre™ group also demonstrated a higher tendency for indwelling bladder catheterization, similar to the urinary retention data observed in literature with Safyre TOT ( 12 , 16 , 21 ). Palma et al. reported urinary retention in 3% of patients after a transobturator Safyre™ sling, which may be treated by loosen the sling tension ( 16 ). We used the retropubic approach, which is known to present higher risk for retention since the sling band can become more compressive around the urethra. In our study, 3 (2.4%) patients in the HMS group and 4 (4.2%) patients in the Safyre™ group required urethrolysis due persistent emptying LUTS.

The importance of reporting the presence of vaginal extrusions has increased in recent years. Several authors who have published studies with homemade slings do not report local complications with the mesh, prioritizing only the reporting of voiding complications ( 8 - 12 ). Vaginal extrusion rates showed a higher tendency in the HMS group. Ciftci et al. reported a higher rate in HMS group (14.6% vs. 1.6%) after 12 months of follow-up ( 8 ), using a transobturator approach, and ElSheemy MS 10% ( 17 ). Other studies with Safyre™ reported extrusion rates between 5 – 8.8% up to 96 months of follow-up ( 13 , 14 , 21 ). Our overall vaginal extrusion rate was 9.9% and the median time to onset of extrusion was 4.5 months. It is difficult to compare these results, since extrusion definition varies in literature, besides vaginal extrusion correlates with several variables such as characteristics of the mesh, follow-up extension, intrinsic patient factors and the surgical route of the sling ( 5 , 6 ). Furthermore, long-term extrusions may not be properly assessed by teleconsultations, especially those asymptomatic patients. Certainly, an in-face clinical visit with a vaginal exam would be better. It is not also possible to draw definitive conclusions regarding this specific complication, as the sample size was not calculated based on the expected complication rate.

Telephone evaluation performed during the study revealed persistence of SUI in 20.7% – 31.3% and urgency incontinence in 40% – 42.4% in the mid and long-term follow-up. Other studies using Safyre™ have found recurrent incontinence ranging from 17.6 – 21% in up to 96 months of follow-up ( 13 , 21 ). Subjective success rate in the long-term follow-up was 68,7% in HMS and 79,3% in the Safyre™ group, which was lower than the reported by Kenton et al. (79% – 85%) or Sahin et al. (88%) after 5 years ( 3 , 22 ). In fact, a decline in the mid- and long-term MUS treatment success has been repeatedly reported ( 23 - 25 ).

In a retrospective study comparing transobturator HMS and commercial slings, Ciftci et al. reported similar subjective cure rates in both groups after a 12-month follow-up ( 8 ) and Lourenço et al. found a comparable rate of subjective cure rates ( 9 ). Other authors found subjective cure rates between 59% and 90% ( 13 , 15 , 21 ). Palma et al., using transobturator Safyre™, observed a subjective cure rate of 90% in the first and sixth postoperative months, reporting that they were able to maintain such results due to the possibility of sling adjustments ( 16 ). Similarly, but using a handmade sling, Toledo et al. demonstrated that it is possible to adjust it, which can prevent immediate failures ( 18 ).

Limitations of this study include the retrospective design, lack of randomization, and use of postoperative telephone calls at different follow-up times. Nevertheless, telephone standardized validated questionnaires helped to reach many patients and has been described as an appropriate follow-up method ( 26 ). Especially after Covid 19 pandemic, telephone assessment has been increasingly accepted, as the telephone evaluation allows a safe and efficacious follow-up for MUS patients ( 27 ).

## CONCLUSION

Patients undergoing SUI surgery using HMS or Safyre™ presented similar satisfaction and subjective cure rates, with no significant differences in quality-of-life on urinary incontinence scores. Higher rates of intraoperative bladder injury were seen in patients who received Safyre™ retropubic sling.
